# Correlation between anticoagulant initiation time and thrombolysis at 30 days after anticoagulation therapy in patients with pulmonary embolism

**DOI:** 10.3389/fmed.2025.1690899

**Published:** 2025-11-18

**Authors:** Yaojie Zhou, Danrong Yang, Yi Liu

**Affiliations:** 1 Shanghai Sixth People's Hospital Affiliated to Jiao Tong University School of Medicine, Shanghai, China; 2 Department of Respiratory and Critical Care Medicine, Shanghai, China

**Keywords:** pulmonary embolism, pulmonary artery obstruction index, anticoagulation, D-dimer, thrombolysis

## Abstract

**Objective:**

This study aimed to investigate the correlation between anticoagulant initiation time and thrombolysis at 30 days after anticoagulation therapy in patients with pulmonary embolism (PE).

**Methods:**

Patients with PE who were hospitalized in the Department of Respiratory Medicine were enrolled. Based on computed tomography pulmonary angiography (CTPA) performed 30 days after anticoagulation therapy, patients were categorized into the complete thrombolysis group (*n* = 112) and the incomplete thrombolysis group (*n* = 93). A retrospective analysis was conducted on risk factors, clinical characteristics, and auxiliary examinations. Spearman's correlation analysis was used to assess the association between anticoagulant initiation time and thrombolysis at 30 days. Receiver operating characteristic (ROC) curve analysis was applied to determine the optimal cut-off point for anticoagulation initiation time in PE.

**Results:**

The proportion of patients with complete thrombolysis at 30 days was 54.63% (112 of 205). Spearman's correlation analysis indicated a significant negative correlation between anticoagulant initiation time and thrombolysis at 30 days (Rs = −0.411, *P* < 0.001). ROC curve analysis showed that anticoagulant initiation time predicted thrombolysis at 30 days (AUC: 0.736, 95% CI: 0.668–0.804, *P* < 0.001). The Youden index was 0.071, and the optimal cut-off point for anticoagulant initiation time was <6.5 days, which was associated with the best therapeutic effect.

**Conclusion:**

Anticoagulant initiation time, PAOI, DVT, and malignant tumors were negatively correlated with thrombolysis at 30 days after anticoagulation therapy. Thrombus absorption within 1 month was significantly improved when anticoagulant therapy was initiated within 6.5 days.

## 1 Introduction

Pulmonary embolism (PE) is the third leading cause of death from cardiovascular disease, following myocardial infarction and stroke (1, 2). Recent studies have shown that the incidence of PE in hospitalized patients in China in 2021 was 14.19 per 100,000, with a slightly higher incidence in men than in women (3). Risk stratification using hemodynamic parameters, biomarkers of myocardial injury, and markers of right ventricular dysfunction assessed by echocardiography or computed tomography is strongly recommended after confirmation of PE, followed by appropriate treatment (4–6). For patients with low-risk PE, anticoagulant therapy may be administered in the outpatient setting, while patients at high risk may require thrombolytic therapy (7, 8).

2019 ESC guidelines for the diagnosis and management of acute pulmonary embolism (9), and 2025 Chinese Society of Cardiology (CSC) guidelines for the diagnosis and management of PE recommend anticoagulant therapy for at least 3 months, with continuation determined by the degree of thrombus absorption (10). Factors influencing thrombolytic absorption include systemic thrombolytic agents, catheter-directed thrombolysis, and interventional therapies in patients with high-risk PE, which may accelerate thrombus resolution (11–13).

In clinical practice, earlier initiation of anticoagulation has been observed to result in better thrombolytic outcomes. However, few studies have investigated the optimal timing of anticoagulant initiation. In this study, we performed a retrospective case–control analysis to evaluate the correlation between anticoagulation initiation time and thrombolysis at 30 days in patients with PE, aiming to provide evidence to guide clinical practice.

## 2 Materials and methods

### 2.1 Study subjects

This study retrospectively collected patients with PE, in the Department of Respiratory and Critical Care Medicine, Shanghai Sixth People's Hospital, between September 1, 2018 and January 30, 2025, were included. The inclusion criteria followed the diagnostic guidelines outlined in the “2019 ESC guidelines for the diagnosis and management of acute pulmonary embolism developed in collaboration with ERS” issued by ESC (9).

Inclusion criteria: (1) Age ≥18 years; (2) first-time diagnosis of PE confirmed by computed tomography pulmonary angiography (CTPA); (3) disease duration < 30 days; (4) no contraindications to anticoagulation therapy; (5) completion of at least 90 days of treatment and follow-up; (6) CTPA performed before anticoagulation therapy and repeated at 30 and 90 days after treatment.

Exclusion criteria: Patients were excluded if they: (1) refused anticoagulation therapy; (2) had severe heart disease, advanced malignant tumor, or an expected survival time < 2 weeks; (3) had other types of embolism such as fat embolism or chronic pulmonary embolism; (4) received thrombolytic therapy; (5) presented with clinically significant thrombocytopenia (platelet count < 75 × 10^9^/L); (6) had coagulation disorders or bleeding tendencies that contraindicated anticoagulation therapy according to the treating physician; (7) were initially diagnosed with PE at another hospital before referral; (8) had incomplete medical records; (9) could not undergo CTPA due to obesity, severe renal insufficiency, or contrast allergy; (10) had contraindications to direct oral anticoagulants (DOACs), such as chronic kidney disease with creatinine clearance < 30 mg/dL, cirrhosis (Child–Pugh class B or C), alanine aminotransferase more than twice the upper normal limit, age < 18 years, or pregnancy.

**Figure 1:**
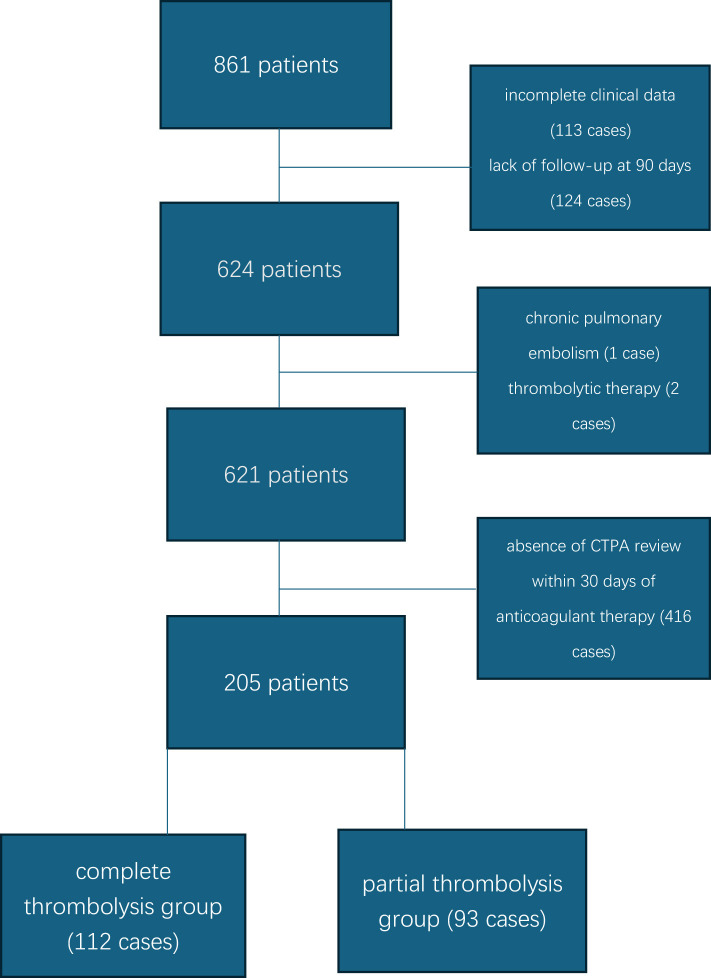
Specific flowchart.

This study was a single center retrospective design. A total of 861 patients with first-ever PE were initially enrolled. After excluding patients with incomplete clinical data (113 cases), absence of CTPA review within 30 days of anticoagulant therapy (416 cases), lack of follow-up at 90 days (124 cases), chronic pulmonary embolism (1 case), and thrombolytic therapy (2 cases), 205 patients were finally included. Based on CTPA findings at 30 days, patients were categorized into the complete thrombolysis group (112 cases) and the partial thrombolysis group (93 cases) (Figure 1).

This study was approved by the Ethics Committee of Shanghai Sixth People's Hospital, Shanghai Jiao Tong University. All procedures involving human participants were conducted in accordance with the ethical standards of the institutional and national research committees, as well as the principles outlined in the 1964 Helsinki Declaration and its subsequent amendments or comparable ethical standards. Due to the retrospective nature of the study, informed consent was waived [No.: 2023-KY-138(K)].

### 2.2 Data collection

The following data were retrospectively collected and analyzed: gender, age, body mass index (BMI), smoking history, underlying diseases (hypertension, stroke, diabetes, recent fracture within the past 4 weeks, recent fixation or surgery, malignancy), major symptoms (chest pain, dyspnea, etc.), arterial blood gas analysis, plasma D-dimer (D-D), N-terminal pro-brain natriuretic peptide (NT-proBNP), cardiac troponin I (cTNI), electrocardiographic findings, pulmonary artery systolic pressure (PASP; measured via echocardiography), lower extremity venous ultrasound findings, thrombus location, pulmonary hypertension (defined as PASP > 40 mmHg), pulmonary vascular obstruction index (PAOI) (14), CSC-defined PE risk categories (low risk, intermediate-low risk, intermediate-high risk, high risk), simplified Pulmonary Embolism Severity Index (sPESI; scores of 0, 1, 2, ≥3) (15), bleeding manifestations, treatment regimens, and outcomes.

### 2.3 Treatment

Patients with PE received anticoagulant therapy in accordance with the “2019 ESC guidelines for the diagnosis and management of acute pulmonary embolism developed in collaboration with ERS” (9).

Following PE diagnosis, anticoagulation was initiated. In 77 patients, warfarin therapy was administered as follows: subcutaneous low-molecular-weight heparin (100 IU/kg every 12 h) was started, and oral warfarin (2.5 mg) was introduced 24–48 h later. Both agents were used concurrently for 4–5 days. When the prothrombin time (PT) and international normalized ratio (INR) reached 2.0–3.0 for two consecutive days, low-molecular-weight heparin was discontinued, while warfarin was continued orally. Warfarin dosage was adjusted as follows: increased by 0.625 mg if INR < 2.0, and reduced or discontinued if INR > 3.0.

In 128 patients, rivaroxaban was used (15 mg orally twice daily for 3 weeks, followed by 20 mg once daily for 9 weeks). All patients received anticoagulant therapy for 3 months. None experienced adverse events such as subcutaneous hematoma, gastrointestinal bleeding, or intracranial hemorrhage.

CTPA was repeated 90 days after anticoagulant therapy to evaluate thrombus resolution in patients of the partial thrombolysis group. In the complete thrombolysis group, serum D-dimer was measured on day 90. If D-dimer was positive, CTPA was performed to confirm the presence of pulmonary thrombosis. If D-dimer was negative, pulmonary thromboembolism was excluded.

### 2.4 Statistical analysis

Statistical analyses were performed using SPSS version 17.0. The Mann–Whitney *U*-test was used for intergroup comparisons. Normally distributed data were expressed as mean ± standard deviation (Mean ± SD) and compared using the *t*-test. Categorical variables were presented as frequencies (%) and compared using the χ^2^ test. Logistic regression analysis was conducted, with thrombolysis at 30 days after anticoagulation therapy (±) as the dependent variable, and gender, age, risk factors, comorbidities, arterial blood gas parameters, D-dimer, cTNI, and NT-proBNP as independent variables to identify predictors of thrombolysis. Spearman's correlation analysis was applied to examine the association between anticoagulant initiation time and thrombolysis at 30 days. Receiver operating characteristic (ROC) curve analysis was performed to determine the optimal cut-off point for anticoagulation initiation time in PE. A *p*-value < 0.05 was considered statistically significant.

## 3 Results

### 3.1 Baseline characteristics

A total of 205 patients with PE, including 89 males and 116 females aged 22–94 years, were included. CTPA was repeated 30 days after initiation of anticoagulant therapy. Based on thrombolysis status, patients were classified into the complete thrombolysis group (112 cases) and the incomplete thrombolysis group (93 cases). The overall rate of thrombolysis at 30 days was 54.63%.

Among the 77 patients treated with warfarin, 44 achieved complete thrombolysis and 33 had incomplete thrombolysis, yielding a thrombolysis rate of 57.14%. Among the 128 patients treated with rivaroxaban, 68 achieved complete thrombolysis and 60 had incomplete thrombolysis, yielding a thrombolysis rate of 53.12%. Comparison between the rivaroxaban and warfarin groups showed no significant difference (χ^2^ = 0.313, *P* = 0.576). Gingival bleeding occurred in three patients receiving warfarin and three patients receiving rivaroxaban. No cases of hemoptysis, subcutaneous hematoma, gastrointestinal bleeding, or intracranial hemorrhage were reported. All patients completed anticoagulant therapy, survived, and were discharged. After 3 months, only one patient in the incomplete thrombolysis group had residual pulmonary thrombus. Detailed results are presented in Tables 1–6.

**Table 1:** Comparison of basic information (mean ± SD) (*n*, %).

**General Information**	**A group (n = 112 cases)**	**B group (n = 93 cases)**	***F*/χ^2^value**	***P*-value**
Male/Female (cases)	50/62	39/55	0.152	0.697
Age (years)	69.20 ± 11.31	70.72 ± 13.49	1.168	0.281
BMI (kg/m^2^)	20.76 ± 2.887	20.99 ± 2.927	0.095	0.759
Smoking history (cases)	44 (39.28%)	30 (32.25%)	1.088	0.297
Symptom duration (days)	4.87 ± 2.191	7.51 ± 3.242	15.529	0.001^*^
Oral contraceptive use (cases)	0	0	–	–
Long-haul travel history (cases)	0	0	–	–
Atrial fibrillation (cases)	7 (6.25%)	3 (3.22%)	0.947	0.324
Hypertension (cases)	56 (50.0%)	50 (53.26%)	0.288	0.591
Diabetes mellitus (cases)	22 (19.6%)	18 (19.35%)	0.003	0.959
Malignant tumor (cases)	4 (3.57%)	18 (19.35%)	13.212	< 0.001^*^
Surgery or trauma (cases)	19 (16.9%)	18 (19.35%)	0.196	0.658
Stroke history (cases)	6 (5.35%)	7 (7.52%)	0.403	0.526
Cough	24 (21.4%)	28 (30.1%)	2.022	0.155
Fever	17 (15.17%)	19 (20.43%)	0.968	0.325
Chest pain	23 (20.53%)	16 (17.2%)	0.366	0.545
Chest distress	44 (39.28%)	35 (37.63%)	0.058	0.809
SBP at admission, (mmHg)	130.45 ± 13.54	133.45 ± 14.32	0.134	0.715
DBP at admission, (mmHg)	80.7 ± 11.09	79.22 ± 10.95	0.002	0.961
Heart rate at admission, (bpm)	91.15 ± 20.04	88.58 ± 19.15	0.520	0.472

^*^P < 0.05.

BMI, body mass index; SBP, Systolic blood pressure; DBP, diastolic blood pressure; bpm, beats per min;

**Table 2:** Comparison of laboratory examinations (mean ± SD) (*n*, %).

**General information**	**A group (*n* = 112 cases)**	**B group (*n* = 93 cases)**	***F*/χ^2^ value**	***P*-value**
sPESI Class (cases)			2.192	0.534
0	34 (30.35%)	25 (26.88%)		
1	49 (43.75%)	44 (47.31%)		
2	24 (21.42%)	16 (17.2%)		
≥3	5 (4.46%)	8 (8.6%)		
cTNI (ug/L)	0.0827 ± 0.166	0.066 ± 0.129	2.326	0.129
Pro-BNP (ng/L)	601.51 ± 796.35	589.88 ± 632.96	0.805	0.371
PaO2 (mmHg)	77.32 ± 17.74	81.13 ± 17.60	1.273	0.261
PaCO2 (mmHg)	37.56 ± 5.85	36.86 ± 5.43	0.014	0.904
D-Dimer (mg/L)	6.95 ± 5.07	6.96 ± 6.07	1.701	0.194
PASP (mmHg)	29.29 ± 11.24	28.01 ± 8.89	2.424	0121
PVOI (%)	25.49 ± 8.90	31.58 ± 11.22	15.65	< 0.001^*^
CTS-defined risk PE			3.657	0.161
Low-risk patients	51 (45.53%)	37 (39.78%)		
Intermediate-low-risk patients	45 (40.17%)	48 (51.61%)		
Intermediate-high-risk patients	17 (15.17%)	8 (8.6%)		
DVT (cases)	19 (16.9%)	39 (41.93%)	15.617	< 0.001^*^
PH (cases)	10 (8.92%)	7 (7.52%)	1.318	0.187
Minor bleeding (cases)	3 (2.67%)	3 (3.22%)	0.054	0.817
Hospitalization duration (days)	10.59 ± 3.43	10.70 ± 4.00	0.045	0.832
90-day mortality (cases)	0	0	–	–

^*^P < 0.05.

TNI, troponin I; BNP, B-type natriuretic peptide; PVOI, pulmonary vascular obstruction index; PaCO_2_, partial pressure of carbon dioxide; PaO_2_, partial pressure of oxygen; DVT, deep vein thrombosis; PASP, pulmonary artery systolic pressure; PH, pulmonary hypertension; sPESI, simplified pulmonary embolism severity index.

**Table 3:** Comparison of basic information (mean ± SD) (*n*, %) (Warfarin group).

**General information**	**A group (*n* = 44 cases)**	**B group (*n* = 33 cases)**	***F*/χ^2^-value**	***P*-value**
Male/female (cases)	14/30	10/23	2.203	0.138
Age (years)	67.34 ± 9.852	70.0 ± 12.62	1.944	0.167
BMI (kg/m^2^)	20.32 ± 2.84	21.53 ± 3.068	0.119	0.731
Smoking history (cases)	15 (34.09%)	10 (30.3%)	0.123	0.725
Symptom duration (days)	4.75 ± 2.334	7.48 ± 3.474	6.474	0.013^*^
Oral contraceptive use (cases)	0	0	–	–
Long-haul travel history (cases)	0	0	–	–
Atrial fibrillation (cases)	3 (6.81%)	1 (3.03%)	0.549	0.459
Hypertension (cases)	26 (59.09%)	17 (51.51%)	0.439	0.508
Diabetes mellitus (cases)	7 (15.9%)	7 (21.21%)	0.356	0.550
Malignant tumor (cases)	1 (2.27%)	9 (27.27%)	10.429	0.001^*^
Surgery or trauma (cases)	7 (15.9%)	6 (18.18%)	0.069	0.792
Stroke history (cases)	2 (4.54%)	2 (6.06%)	0.088	0.767
Cough	10 (22.72%)	11 (33.3%)	1.069	0.301
Fever	10 (22.72%)	11 (33.3%)	0.716	0.397
Chest pain	9 (20.45%)	7 (21.21%)	0.007	0.935
Chest distress	23 (52.27%)	11 (33.3%)	2.743	0.098
SBP at admission, (mmHg)	130.45 ± 13.54	133.45 ± 14.32	0.134	0.715
DBP at admission, (mmHg)	75.59 ± 10.23	74.52 ± 8.74	0.925	0.339
Heart rate at admission, (bpm)	86.55 ± 18.64	88.85 ± 18.44	0.095	0.759

^*^P < 0.05.

BMI, body mass index; SBP, systolic blood pressure; DBP, diastolic blood pressure; bpm, beats per minute.

**Table 4:** Comparison of laboratory examinations (mean ± SD) (*n*, %) (Warfarin group)

**General information**	**A group (*n* = 44 cases)**	**B group (*n* = 33 cases)**	***F*/χ^2^ value**	***P*-value**
sPESI Class (cases)			3.631	0.304
0	16 (36.36%)	9 (27.27%)		
1	21 (47.72%)	13 (39.39%)		
2	6 (13.63%)	8 (24.24%)		
≥3	1 (2.27%)	3 (9.09%)		
cTNI (ug/L)	0.0636 ± 0.117	0.0681 ± 0.157	0.447	0.506
Pro-BNP (ng/L)	531.18 ± 679.06	442.30 ± 603.39	1.014	0.317
PaO2 (mmHg)	77.186 ± 19.768	79.08 ± 17.20	0.002	0.962
PaCO2 (mmHg)	38.30 ± 6.40	36.17 ± 6.12	0.086	0.770
D-Dimer (mg/L)	6.69 ± 4.92	6.186 ± 4.59	0.026	0.873
PASP (mmHg)	28.82 ± 9.61	29.09 ± 10.99	0.233	0.631
PVOI (%)	23.125 ± 8.76	31.894 ± 12.516	9.44	0.003^*^
CTS-defined risk PE			0.379	0.927
Low-risk patients	23 (52.25%)	18 (54.5%)		
Intermediate-low-risk patients	15 (34.09%)	12 (36.36%)		
Intermediate-high-risk patients	6 (13.63%)	3 (9.09%)		
DVT (cases)	7 (15.9%)	14 (42.42%)	6.684	0.010^*^
PH (cases)	5 (11.36%)	4 (12.12%)	0.01	0.918
Minor bleeding (cases)	2 (4.54%)	1 (3.03%)	0.116	0.734
Hospitalization duration (days)	9.86 ± 3.001	9.94 ± 2.263	1.804	0.183
90-day mortality (cases)	0	0	–	–

^*^P < 0.05.

TNI, troponin I; BNP, B-type natriuretic peptide; PVOI, pulmonary vascular obstruction index; PaCO_2_, partial pressure of carbon dioxide; PaO_2_, partial pressure of oxygen; DVT, deep vein thrombosis; PASP, pulmonary artery systolic pressure; PH, pulmonary hypertension; sPESI, simplified pulmonary embolism severity index.

**Table 5:** Comparison of basic information (mean ± SD) (*n*, %) (Rivaroxaban group).

**General information**	**A group (*n* = 68 cases)**	**B group (*n* = 60cases)**	***F*/χ^2^-value**	***P*-value**
Male/female (cases)	36/33	23/37	2.477	0.116
Age (years)	70.40 ± 12.08	71.12 ± 14.03	0.328	0.568
BMI (kg/m^2^)	21.04 ± 2.90	20.70 ± 2.83	0.607	0.437
Smoking history (cases)	29 (42.64%)	20 (33.33%)	1.17	0.279
Symptom duration (days)	4.94 ± 2.108	7.52 ± 3.138	5.047	0.026^*^
Oral contraceptive use (cases)	0	0	–	–
Long-haul travel history (cases)	0	0	–	–
Atrial fibrillation (cases)	4 (5.88%)	2 (3.33%)	0.464	0.496
Hypertension (cases)	30 (44.11%)	33 (55.0%)	1.51	0.219
Diabetes mellitus (cases)	15 (22.05%)	11 (18.33%)	0.320	0.572
Malignant tumor (cases)	3 (4.41%)	9 (15.0%)	4.206	0.040^*^
Surgery or trauma (cases)	12 (17.64%)	13 (21.66%)	0.328	0.567
Stroke history (cases)	4 (5.88%)	5 (8.33%)	0.464	0.496
Cough	14 (20.58%)	17 (28.33%)	1.042	0.307
Fever	12 (17.64%)	13 (21.66%)	0.328	0.567
Chest pain	14 (20.58%)	9 (15.0%)	0.675	0.411
Chest distress	21 (30.88%)	24 (40.0%)	1.162	0.281
SBP at admission, (mmHg)	130.22 ± 12.64	134.82 ± 14.42	1.104	0.295
DBP at admission, (mmHg)	84.0 ± 10.41	81.80 ± 11.24	0.573	0.450
Heart rate at admission, (bpm)	94.13 ± 20.48	88.43 ± 19.68	0.938	0.335

^*^P < 0.05.

BMI, body mass index; SBP, systolic blood pressure; DBP, diastolic blood pressure; bpm, beats per minute.

**Table 6:** Comparison of laboratory examinations (mean ± SD) (*n*, %) (Rivaroxaban group).

**General information**	**A group (*n* = 68 cases)**	**B group (*n* = 60 cases)**	***F*/χ^2^ value**	***P*-value**
sPESI Class (cases)			3.742	0.291
0	18 (26.47%)	16 (26.67%)		
1	28 (41.17%)	31 (51.66%)		
2	18 (26.47%)	8 (13.33%)		
≥3	4 (5.88%)	5 (8.33%)		
cTNI (ug/L)	0.095 ± 0.191	0.065 ± 0.113	5.921	0.016^*^
Pro-BNP (ng/L)	647.01 ± 865.65	671.05 ± 639.05	0.701	0.404
PaO2 (mmHg)	77.41 ± 16.46	82.25 ± 17.86	2.207	0.140
PaCO2 (mmHg)	37.07 ± 5.467	37.25 ± 5.01	0.045	0.833
D-Dimer (mg/L)	7.124 ± 5.192	7.389 ± 6.74	1.718	0.192
PASP (mmHg)	29.60 ± 12.23	27.82 ± 7.969	5.369	0.121
PVOI (%)	27.022 ± 8.716	31.417 ± 10.55	5.184	0.024^*^
CTS-defined risk PE			3.701	0.157
Low-risk patients	27 (39.7%)	19 (31.67%)		
Intermediate-low-risk patients	30 (44.11%)	36 (60.0%)		
Intermediate-high-risk patients	11 (16.17%)	5 (8.33%)		
DVT (cases)	12 (17.64%)	25 (41.67%)	8.949	0.003^*^
PH (cases)	9 (13.23%)	6 (10.0%)	0.322	0.570
Minor bleeding (cases)	1 (1.47%)	2 (3.33%)	0.483	0.487
Hospitalization duration (days)	11.06 ± 3.636	11.12 ± 4.658	0.808	0.371
90-day mortality (cases)	0	0	-	-

^*^P < 0.05.

TNI, troponin I; BNP, B-type natriuretic peptide; PVOI, pulmonary vascular obstruction index; PaCO_2_, partial pressure of carbon dioxide; PaO_2_, partial pressure of oxygen; DVT, deep vein thrombosis; PASP, pulmonary artery systolic pressure; PH, pulmonary hypertension; sPESI, simplified pulmonary embolism severity index.

### 3.2 Univariate and multivariate logistic regression

Univariate logistic regression analysis revealed that thrombolysis at 30 days after anticoagulation therapy was associated with malignant tumor (*F* = 13.212, *P* < 0.001), deep vein thrombosis (DVT) (*F* = 15.617, *P* < 0.001), PAOI (*F* = 15.659, *P* < 0.001), and anticoagulant initiation time (*F* = 10.529, P = 0.001). Variables with statistical significance in univariate analysis were included in multivariate logistic regression. The results indicated that thrombolysis at 30 days after anticoagulation therapy was independently associated with malignant tumors (*B* = −2.253, Wald = 9.934, OR = 0.105, 95% CI: 0.026–0.427, *P* = 0.002), anticoagulant initiation time (*B* = −0.434, Wald = 29.009, OR = 0.648, 95% CI: 0.553–0.759, *P* < 0.001), DVT (*B* = −1.465, Wald = 13.339, OR = 0.231, 95% CI: 0.105–0.507, *P* < 0.001), and PAOI (*B* = −0.059, Wald = 10.338, OR = 0.942, 95% CI: 0.909–0.977, *P* = 0.001).

In the rivaroxaban group, univariate logistic regression analyses were conducted to evaluate the associations between clinical variables and thrombolysis at 30 days after anticoagulation therapy in patients with PE. The results indicated that thrombolysis at 30 days after anticoagulation therapy was associated with DVT (*F* = 8.949, *P* = 0.003), PAOI (*F* = 5.184, *P* = 0.024), TNI (*F* = 5.921, *P* = 0.016), PASP (*F* = 5.369, *P* = 0.022), anticoagulant initiation time (*F* = 5.047, *P* = 0.026), and malignancy (*F* = 4.206, *P* = 0.040). Multivariate logistic regression analysis was performed for variables that showed statistically significant associations in the univariate analysis. It revealed that thrombolysis at 30 days after anticoagulation therapy was independently associated with PAOI (*B* = −0.057, Wald = 5.506, OR = 0.945, 95% CI: 0.901–0.991, *P* = 0.019), malignancy (*B* = −1.906, Wald = 4.259, OR = 0.149, 95% CI: 0.024–0.909, *P* = 0.039), anticoagulant initiation time (*B* = −0.441, Wald = 16.428, OR = 0.644, 95% CI: 0.520–0.797, *P* < 0.001), and DVT (*B* = −1.287, Wald = 6.954, OR = 0.276, 95% CI: 0.106–0.719, *P* = 0.008).

In the warfarin group, univariate logistic regression analyses were conducted to evaluate the associations between clinical variables and thrombolysis at 30 days after anticoagulation therapy in patients with PE. The results indicated that thrombolysis at 30 days after anticoagulation therapy was associated with malignancy (*F* = 6.474, *P* = 0.011), DVT (*F* = 6.684, *P* = 0.010), PAOI (*F* = 9.440, *P* = 0.003), and anticoagulant initiation time (*F* = 6.474, *P* = 0.013). Multivariate logistic regression analysis was performed for variables that showed statistically significant associations in the univariate analysis. It revealed that thrombolysis at 30 days after anticoagulation therapy was independently associated with anticoagulant initiation time (*B* = −0.427, Wald = 11.346, OR = 0.653, 95% CI: 0.509–0.837, *P* = 0.001), malignancy (*B* = −3.780, Wald = 5.183, OR = 0.023, 95% CI: 0.001–0.591, *P* = 0.023), PAOI (*B* = −0.074, Wald = 5.237, OR = 0.929, 95% CI: 0.872–0.989, *P* = 0.022), and DVT (*B* = −1.662, Wald = 4.991, OR = 0.190, 95% CI: 0.044–0.816, *P* = 0.025).

### 3.3 Spearman correlation analysis

A weak negative correlation was observed between anticoagulant initiation time and thrombolysis at 30 days after anticoagulation therapy in patients with PE (Rs = −0.411, *P* < 0.001). Additionally, a weak negative correlation was found between PAOI and thrombus disappearance at 30 days (Rs = −0.286, *P* < 0.001).

In the rivaroxaban group, a weak negative correlation was noted between anticoagulant initiation time and thrombolysis at 30 days after anticoagulation therapy (Rs = −0.425, *P* < 0.001), as well as between PAOI and thrombus disappearance at 30 days (Rs = −0.228, *P* < 0.001).

In the warfarin group, a weak negative correlation was observed between anticoagulant initiation time and thrombolysis at 30 days after anticoagulation therapy (Rs = −0.390, *P* < 0.001), and between PAOI and thrombus disappearance at 30 days (Rs = −0.360, *P* = 0.001).

### 3.4 ROC curve analysis

**Figure 2:**
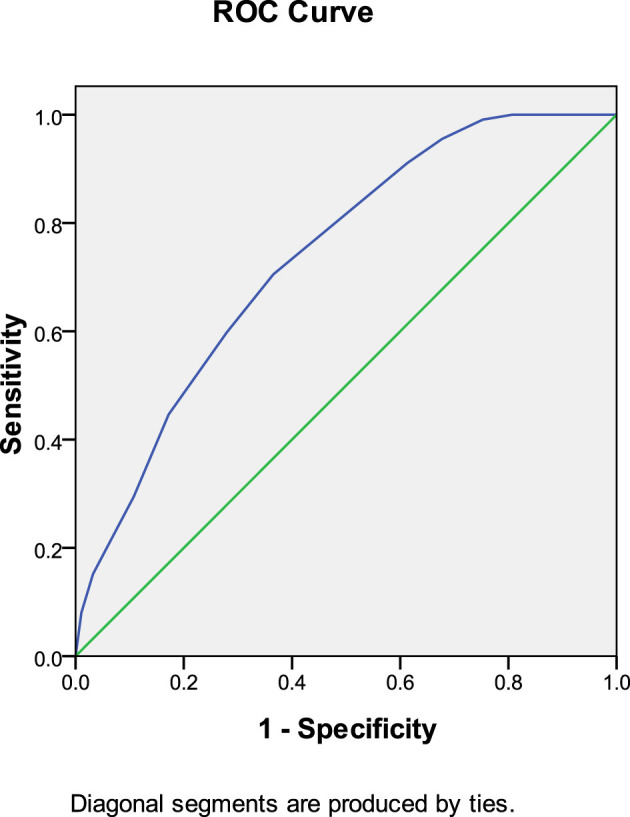
Anticoagulant initiation time predicted thrombolysis at 30 days after anticoagulation therapy.

ROC curve analysis demonstrated that anticoagulant initiation time predicted thrombolysis at 30 days after anticoagulation therapy (AUC: 0.736, 95% CI: 0.668–0.804, *P* < 0.001). The Youden index was 0.071, and an anticoagulation initiation time of < 6.5 days yielded the most favorable therapeutic effect (Figure 2).

**Figure 3:**
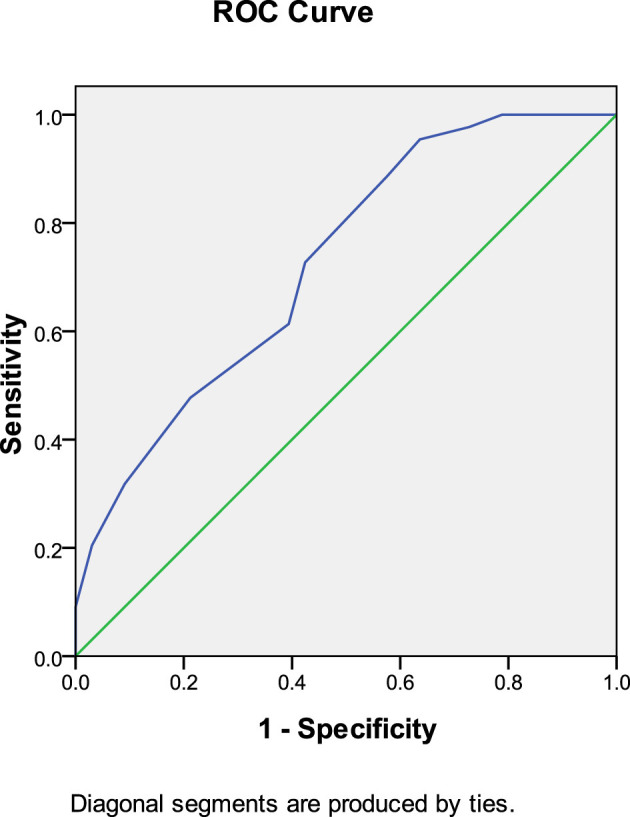
Anticoagulant initiation time predicted thrombolysis at 30 days after anticoagulation therapy (rivaroxaban group).

In the rivaroxaban group, anticoagulant initiation time predicted thrombolysis at 30 days after anticoagulation therapy (AUC: 0.743, 95% CI: 0.657–0.829, *P* < 0.001). The Youden index was 0.024, and an anticoagulation initiation time of < 5.5 days provided the best therapeutic outcome (Figure 3).

**Figure 4:**
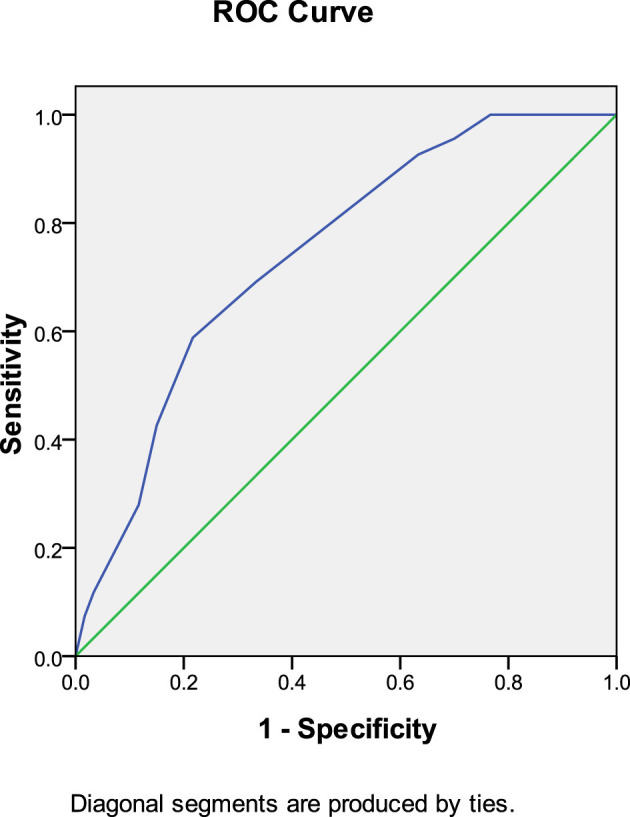
Anticoagulant initiation time predicted thrombolysis at 30 days after anticoagulation therapy (warfarin group).

In the warfarin group, anticoagulant initiation time predicted thrombolysis at 30 days after anticoagulation therapy (AUC: 0.726, 95% CI: 0.612–0.840, *P* = 0.001). The Youden index was 0.591, and an anticoagulation initiation time of < 8.5 days produced the most favorable therapeutic effect (Figure 4).

## 4 Discussion

The ESC guidelines for the diagnosis and management of PE (2019) strongly recommend anticoagulant therapy for 3–6 months, after which the decision to extend treatment should be based on the degree of thrombolysis and absorption (9). However, in most patients with PE, the thrombus is almost completely absorbed within this period, making it difficult to assess the therapeutic effect in time. Repeated CTPA at 30 days after treatment can provide timely evaluation of thrombus dissolution and absorption. For thrombi that are not completely dissolved, interventional therapy can be implemented promptly to prevent thrombus organization and the development of chronic thrombosis. In patients with completely resolved PE, anticoagulant drugs may be adjusted to prophylactic doses according to transient VTE risk factors. Therefore, CTPA re-examination was performed at 30 days after treatment in this study.

The results showed that the rate of complete thrombus dissolution and absorption was 54.63% at 30 days after anticoagulant therapy, indicating that a high proportion of thrombi were dissolved within this period. However, there was no significant difference between the warfarin and rivaroxaban groups. These results were consistent with previous studies (16–19).

Multiple factors may influence thrombus dissolution and absorption. Jervan et al. (20) found that increased thrombus burden at diagnosis (OR 2.08, 95% CI: 1.06–4.06, *P* = 0.032) and PTE without clear predisposing factors (OR 2.25, 95% CI: 1.13–4.48, *P* = 0.021) were associated with an increased risk of residual pulmonary defects. Wang et al. (21) demonstrated that independent factors associated with residual thrombosis included unprovoked PE (OR 0.231, 95% CI: 0.062–0.861) and acute fibrinogen level (OR 1.958, 95% CI: 1.282–2.911). Fang et al. (22) reported that malignancy may affect thrombus absorption. Yu et al. (23) showed that thrombus density ≥61.8 HU and thrombus volume ≥14.0 cm3 were critical thresholds for predicting PE resolution. Kato et al. (24) identified a history of venous thromboembolism or active malignancy as risk factors for residual thrombus. An et al. (25) found that higher PVOI was associated with residual thrombus (*P* = 0.004) and recurrence (*P* = 0.03).

In the above studies, the observation time point was mostly 3–6 months after anticoagulant therapy. In contrast, the present study evaluated the thrombolytic effect at 1 month after anticoagulant therapy. Logistic regression analysis demonstrated that anticoagulant initiation time, malignancy, DVT, and PAOI were significantly correlated with pulmonary thrombolysis.

There have been few studies on the correlation between anticoagulant initiation time and thrombolysis, and no comparisons have been made regarding the initiation times of different anticoagulants. Aranda et al. (26) reported that COPD [OR, 3.22 (1.35–7.71); *p* = 0.009], secondary PE [OR, 2.02 (1.08–3.79); *p* = 0.028], anticoagulant initiation within 7 days [OR, 2.42 (1.22–4.78); *p* = 0.011], and a Qanadli score < 16% [OR, 2.12 (1.03–4.37); *p* = 0.043] were associated with thrombolysis. In another study, Aranda C et al. (27) found that in idiopathic PE, a delay of more than 7 days between symptom onset and diagnosis was associated with residual thrombus. Spearman correlation analysis demonstrated a negative correlation between anticoagulant initiation time and pulmonary thrombolysis (Rs = −0.411, *p* < 0.001). ROC curve analysis indicated that anticoagulant initiation within 6.5 days after symptom onset was associated with a more favorable thrombolytic effect within 1 month. The average anticoagulation initiation time was 8.5 days for warfarin and 5.5 days for rivaroxaban. The possible reasons for this are as follows: (1) rivaroxaban has been widely adopted in recent years, and with increased clinical awareness of PE, earlier and more rapid diagnoses have been achieved, leading to significantly shorter anticoagulant initiation times and improved treatment efficacy; (2) earlier initiation of anticoagulation allows treatment of relatively fresh thrombi, thereby accelerating thrombus dissolution and improving therapeutic outcomes. Mansueto et al. (28) observed histologically that platelet aggregation occurred within 1 h after thrombus formation by analyzing autopsy specimens of pulmonary thrombosis. Lymphocytes appeared between 1 and 24 h after thrombus formation. Between 48 and 72 h, fibroblasts were observed at the thrombus periphery, and after 72 h, numerous fibroblasts and collagen fibers were present within the thrombus. Therefore, earlier initiation of anticoagulation yields a more favorable therapeutic effect.

In this study, we found that patients in the incomplete thrombolysis group may have been influenced by the following factors: (1) some patients exhibited a larger PAOI, often associated with a substantial thrombus burden in the main pulmonary artery. In addition, some patients presented with DVT, resulting in a greater thrombus load, and consequently, thrombus dissolution and absorption proceeded relatively slowly during the 30 days following anticoagulation; (2) some patients had malignant tumors, and cancer-related thrombus could not be excluded, which may have further hindered thrombus dissolution and absorption. In this cohort, one patient still presented with thrombosis 90 days after anticoagulant therapy, raising the possibility of tumor thrombus; (3) some patients were elderly. In these patients, age-related declines in systemic and vascular function likely impaired thrombus dissolution and absorption.

However, this study has certain limitations. First, its retrospective design at a single center limits the representativeness of the sample. According to PE guidelines, CTPA should be performed at 3–6 months after anticoagulant therapy, whereas in this study, CTPA was performed at 30 days, resulting in a relatively lower detection rate. Second, PAOI was chosen as the index for evaluating thrombus occlusion, which does not precisely quantify thrombus volume. This may have affected the assessment of thrombus dissolution and absorption after anticoagulation therapy. Third, in accordance with PE guidelines, CTPA was used as the primary diagnostic tool in this study. Nevertheless, the sensitivity of CTPA for detecting small distal thrombi is lower than that of radionuclide pulmonary ventilation/perfusion (V/Q) imaging, which may have influenced the results of this study.

## 5 Conclusions

In summary, anticoagulant initiation time, malignant tumors, DVT, and PAOI were factors influencing thrombus dissolution and absorption. When anticoagulation was initiated within 6.5 days, thrombi were largely absorbed within 1 month. However, when anticoagulant initiation time exceeded 6.5 days, thrombus absorption after anticoagulation was delayed. Therefore, early diagnosis and early initiation of anticoagulation are essential for achieving optimal thrombolytic outcomes.

## Data Availability

The raw data supporting the conclusions of this article will be made available by the authors, without undue reservation.
